# Haplotypic and Genotypic Association of Catechol-*O*-Methyltransferase rs4680 and rs4818 Polymorphisms and Treatment Resistance in Schizophrenia

**DOI:** 10.3389/fphar.2018.00705

**Published:** 2018-07-03

**Authors:** Marina Sagud, Lucija Tudor, Suzana Uzun, Matea Nikolac Perkovic, Maja Zivkovic, Marcela Konjevod, Oliver Kozumplik, Bjanka Vuksan Cusa, Dubravka Svob Strac, Iva Rados, Ninoslav Mimica, Alma Mihaljevic Peles, Gordana Nedic Erjavec, Nela Pivac

**Affiliations:** ^1^School of Medicine, University of Zagreb, Zagreb, Croatia; ^2^Department of Psychiatry, University Hospital Centre Zagreb, Zagreb, Croatia; ^3^Laboratory for Molecular Neuropsychiatry, Division of Molecular Medicine, Rudjer Boskovic Institute, Zagreb, Croatia; ^4^Department of Biological Psychiatry and Psychogeriatrics, University Psychiatric Hospital Vrapce, Zagreb, Croatia; ^5^School of Medicine, Josip Juraj Strossmayer University of Osijek, Osijek, Croatia; ^6^Department of Integrative Psychiatry, University Psychiatric Hospital Vrapce, Zagreb, Croatia; ^7^Department of Psychological Medicine, University Hospital Centre Zagreb, Zagreb, Croatia

**Keywords:** treatment-resistant schizophrenia, *COMT* rs4680 polymorphism, *COMT* rs4818 polymorphism, *COMT* rs4680 and rs4818 haplotype, sex-specific association

## Abstract

Treatment-resistant schizophrenia (TRS) continues to be a challenge. It was related to different factors, including alterations in the activity of brain dopaminergic system, which could be influenced by the dopamine-degrading enzyme, catechol-*O*-methyltransferase (COMT). Variants of the *COMT* gene have been extensively studied as risk factors for schizophrenia; however, their association with TRS has been poorly investigated. The aim of the present study was to determine the haplotypic and genotypic association of *COMT* rs4680 and rs4818 polymorphisms with the presence of TRS. Overall, 931 Caucasian patients diagnosed with schizophrenia (386 females and 545 males) were included, while 270 participants met the criteria for TRS. In males, no significant haplotypic and genotypic associations between *COMT* rs4680 and rs4818 polymorphisms and TRS were detected. However, genotypic analyses demonstrated higher frequency of *COMT* rs4680 AA genotype carriers compared to G-allele carriers (*p* = 0.033) and higher frequency of *COMT* rs4818 CC genotype carriers than G-allele carriers (*p* = 0.014) in females with TRS. Haplotype analyses confirmed that the presence of the G allele in females was associated with lower risk of TRS. In women with TRS, the high activity G-G/G-G haplotype was rare, while carriers of other haplotypes were overrepresented (*p* = 0.009). Such associations of *COMT* rs4680 and rs4818 high-activity (G variants), as well as G-G/G-G haplotype, with the lower risk of TRS in females, but not in males, suggest significant, but sex-specific influence of *COMT* variants on the development of treatment-resistance in patients with schizophrenia. However, due to relatively low number of females, those findings require replication in a larger sample.

## Introduction

Schizophrenia is a severe psychiatric disorder. Antipsychotics are the first-line agents in the treatment of schizophrenia, but the clinical response is highly variable. Between 23% (Demjaha et al., 2017) and 47% (Vlatkovic et al., 2018) of patients met the criteria for treatment-resistant schizophrenia (TRS), although the definition has varied across different studies (Howes et al., 2017). In spite of more than 60 years of the widespread use of antipsychotics, TRS continues to present an enormous challenge (Šagud, 2015; Lally et al., 2016). While altered dopaminergic function is the main feature of schizophrenia (Lally et al., 2016; Nedic Erjavec et al., 2017; Nikolac Perkovic et al., 2017; Pruessner et al., 2017), patients with TRS might have distinct dopamine changes, such as lower dopamine synthesis capacity in the striatum (Kim et al., 2017), lower density of dopaminergic synapses in the caudate nucleus (Roberts et al., 2009), and a decrease in the dopamine transporter protein expression (Purves-Tyson et al., 2017), compared to patients who responded to antipsychotics. There is an urgent need to distinguish TRS from non-TRS using genetic or other markers (Šagud, 2015; Lally et al., 2016; Gillespie et al., 2017) as early as possible, in order to provide the best possible treatment for an individual patient.

Catechol-*O*-methyltransferase (COMT) is an important enzyme that degrades catecholamines including dopamine. COMT regulates dopamine availability primarily in the prefrontal cortex (PFC), where the presence of dopamine transporters is scarce (Bilder et al., 2004). Variants of the *COMT* gene have been extensively studied as risk factors for schizophrenia (Egan et al., 2001). Among various polymorphisms of the *COMT* gene, rs4680 and rs4818 significantly affect COMT activity and therefore prefrontal dopamine levels and function. The rs4680 (A > G substitution) or Val108/158Met is the most common functional *COMT* polymorphism in which a G/A substitution results in valine (Val) to methionine (Met) replacement at codon 158 for membrane-bound (MB) COMT, and at codon 108 for the soluble (S) COMT. The Met (A) variant has been associated with a lower thermostability, fourfold lower functional enzyme activity (Lachman et al., 1996), lower protein expression (Chen et al., 2004), and higher dopamine activity compared to Val (G) variant. Previously observed association between *COMT* rs4680 and schizophrenia (Egan et al., 2001; González-Castro et al., 2016) was not confirmed in a meta-analysis (Munafo et al., 2005), or in a cohort with large number of ethnically homogeneous Caucasians in our previous study (Nikolac et al., 2013). Another frequently studied polymorphism of the *COMT* gene is a synonymous polymorphism rs4818, with a C/G substitution (Leu/Leu) at codon 86 of S-COMT or at codon 136 of MB-COMT (Roussos et al., 2008). The G variant of the *COMT* rs4818 polymorphism is associated with greater COMT activity and therefore lower prefrontal dopamine activity (Roussos et al., 2008). It has been reported that *COMT* rs4818 is responsible for the larger variation in the COMT activity than the *COMT* rs4680 polymorphism (Nackley et al., 2006). Some studies demonstrated that *COMT* rs4818 polymorphism was not associated with schizophrenia (Chen C.Y. et al., 2011; Li et al., 2012); however, it is transmitted together with *COMT* rs4680 polymorphism in a haploblock (Hirasawa-Fujita et al., 2018). Contradictory findings exist for the association of *COMT* haplotypes and schizophrenia or its symptoms (Chen C.Y. et al., 2011; Li et al., 2012). Haplotype including *COMT* rs4818 G allele (with rs740603/G allele) was linked to negative symptoms of schizophrenia (Li et al., 2012). However, no significant association of several functional *COMT* polymorphisms and haplotypes with schizophrenia or with psychopathological symptoms was found (Chen C.Y. et al., 2011). Moreover, there were no differences between patients with and without TRS in the whole-blood gene expression of 13 genes, including *COMT* gene (Moretti et al., 2018).

Despite a considerable amount of research on the association of *COMT* rs4680 variants with the response to antipsychotics (Huang et al., 2016), only four studies addressed this *COMT* polymorphism in relation to TRS (Inada et al., 2003; Bosia et al., 2015; Terzić et al., 2016; Rajagopal et al., 2018). These studies yielded inconsistent results, had relatively small sample sizes and the data were not separately analyzed for males and females, although sex-specific associations with *COMT* rs4680 have been reported. For example, the overexpression of the rs4680 GG genotype was found in Spanish males with schizophrenia compared to general population, with no such differences in females (Hoenicka et al., 2010). The presence of one or two A allele in the *COMT* rs4680 elevated the risk of violence in male, but not female patients with schizophrenia (Singh et al., 2012). On the other hand, when *COMT* rs4680 was investigated in Slovenian suicide victims, the AA genotype was more common in the group of control males than in males who committed suicide, and in the control males versus males who committed suicide with violent methods, while again no differences were observed among females (Pivac et al., 2011). The interpretation of the influence of this *COMT* functional polymorphism in psychiatric disorders is therefore complicated by sex-, but also by ethnic-related differences in allele distributions. Whereas Caucasians had similar frequencies of the A and G alleles, the G allele was more common in Asian and other populations (Palmatier et al., 1999). In contrast to these findings, the A allele was associated with bipolar disorder in Asian but not in Caucasian subjects (Taylor, 2018). Moreover, in the meta-analysis of case-control studies, the presence of the G allele of the *COMT* rs4680 was associated with schizophrenia in Caucasian, but not in Asian population, although the data were not analyzed by gender (González-Castro et al., 2016). In addition, only a few studies investigated *COMT* rs4818 polymorphism and the response to antipsychotics (Gupta et al., 2009; Xu et al., 2015; Shi et al., 2017).

Since there is inconclusive or insufficient evidence on the association of *COMT* rs4680 and rs4818 polymorphisms with TRS, especially regarding gender and ethnic differences, the aim of this study was to evaluate genotypic and haplotypic association of the *COMT* rs4680 and rs4818 and TRS in ethnically homogeneous Caucasian subjects of both sexes. Our hypothesis was that patients with TRS have the overrepresentation of rs4680 A allele, as well as rs4818 C allele, compared to non-TRS patients, and that the observed associations with *COMT* are gender-specific.

## Materials and Methods

### Subjects

This cross-sectional study included 931 biologically unrelated Caucasian patients with schizophrenia, 585 males and 386 females, who gave their consent to participate and met the inclusion criteria, and were considered eligible. Subjects were recruited from the University Hospital Centre Zagreb, University Psychiatric Hospital Vrapce, Zagreb, and Neuropsychiatric Hospital Dr. Ivan Barbot, Popovaca, Croatia. Inclusion criteria were in- and out-patients aged 18–65 years, diagnosed with schizophrenia for at least 5 years. Diagnosis was confirmed using the Structured Clinical Interview (SCID; First et al., 1995) based on the DSM-IV criteria [American Psychiatric Association [APA], 1994]. Exclusion criteria were intellectual disabilities, patients with first-episode psychosis and/or no previous treatment with antipsychotics, substance abuse and dependence in the previous three months, any comorbid severe somatic or neurological disorder and patients who had no available detailed medical records with complete psychiatric medication history. After inclusion, all patients underwent complete diagnostic evaluation. Clinical Global Impression-Severity (CGI-S) scale was used to assess the severity of patients’ clinical condition (Guy, 1976). Patients were evaluated using structured interview for the Positive and Negative Syndrome Scale (PANSS) including the PANSS positive, PANSS negative and PANSS general psychopathology subscale (Kay et al., 1987). Schizophrenic patients were subdivided into 574 smokers (i.e., current smokers) and 354 non-smokers (i.e., never smokers and former smokers), whereas for three patients smoking status was not defined. Besides nicotine dependence, no other co-morbid substance abuse or dependence was present. All patients were Caucasians of Croatian origin. The patients were treated with different antipsychotic medication: olanzapine, either as monotherapy or antipsychotic combination (5–20 mg/day), clozapine (300–800 mg/day), risperidone (2–6 mg/day), fluphenazine (5–15 mg/day), haloperidol (4–15 mg/day), promazine (400–500 mg/day), alone or combined with benzodiazepines, i.e., diazepam (2–10 mg/day). Mean dose of antipsychotic medication, calculated into chlorpromazine equivalent doses, was 309.5 ± 263.5 mg/day (range 50–1600 mg/day). Although the concept of TRS is widely used, there is a lack of consensus how to define it. In our study, patients were classified in TRS or non-TRS group according to criteria proposed by Suzuki et al. (2012), which refer to the failure of at least two antipsychotics, given at ≥600 mg chlorpromazine equivalents (Inada and Inagaki, 2015) for more than consecutive 6 weeks, assessed retrospectively. Out of 931 patients, 270 of them met the criteria for TRS and 661 were non-TRS patients. At the time of assessment, psychiatrists were not aware of the genetic test results. The study was approved by the Ethics Committees of the University Hospital Centre Zagreb, University Psychiatric Hospital Vrapce, and Neuropsychiatric Hospital Dr. Ivan Barbot, Popovaca, Croatia, and was carried out in accordance with the Helsinki declaration (1975), as revised in 1983. All patients have signed informed consent prior to study procedures.

### Genotyping

The *COMT* rs4680 (assay ID: C_25746809_50) and rs4818 (assay ID: C_2538750_10) genotypes were determined using DNA isolated from the blood samples with a salting out method (Miller et al., 1988). Genotyping was performed using the primers and probes from the TaqMan^®^ Drug Metabolism Genotyping Assays (Applied Biosystems, Foster City, CA, United States) on ABI Prism 7300 Real time PCR System apparatus (Applied Biosystems, Foster City, CA, United States), according to the procedures described by Applied Biosystems. The 10 μL reaction volume contained 30–100 ng of DNA. Around 10% of randomly selected samples were genotyped again as a quality control for genotyping assays.

### Statistical Analyses

Data were analyzed using Graph Prism version 7.00 (GraphPad Software, Inc.). Data distribution normality was determined with the Kolmogorov–Smirnov normality test. Due to the lack of a normal distribution, Kruskal–Wallis analysis of variance (ANOVA) and Dunn *post hoc* were used to assess differences in age, chlorpromazine equivalent doses, PANSS total, positive, negative, and general psychopathology scores between different groups of patients. The Hardy–Weinberg equilibrium (HWE) was determined using χ^2^-test (Rodriguez et al., 2009). Smoking status, as well as genotype and haplotype distributions, between male and female patients with TRS and non-TRS, were also compared using χ^2^-test (Rodriguez et al., 2009). To assess which category was a major contributor to rejecting the null hypothesis, standardized residuals (*R*; Field et al., 2012) were calculated. Haploview software v. 4.2 (Barrett et al., 2005) was used to determine LD pairwise values for *COMT* rs4818 and rs4680. Loci are considered to be in linkage disequilibrium if *D*′ coefficient is >0.80. Best-estimate haplotype pair for every patient was assigned by PLINK v. 1.07 software using the expectation–maximization algorithm (Purcell et al., 2007). Besides genotypic and haplotypic analyses, additional genetic models (González-Castro et al., 2016) were evaluated: dominant model (G carriers, i.e., GG + GA vs. AA) and recessive model (A carriers, i.e., GA + AA vs. GG) for the *COMT* rs4680, as well as dominant model (C carriers, i.e., CC + CG vs. GG) and recessive model (G carriers, i.e., GG + GC vs. CC) for the *COMT* rs4818. For individual SNP analysis the *p*-value (0.05/2 = 0.025) was corrected because two SNPs were compared and the results were considered significant if *p* < 0.025. G^∗^Power 3 Software (Faul et al., 2009) was used to determine a priori sample size. For a χ^2^-test [with α = 0.025; with expected small effect size = 0.2; power (1 - β) = 0.800], the required sample size was *N* = 288 with *df* = 2; or *N* = 238 for *df* = 1. For Kruskal–Wallis ANOVA, the *p*-value (0.05/4 = 0.0375) was corrected because of four groups into α = 0.0375; with expected small effect size = 0.15; and power (1 - β) = 0.800]; the required sample size was 528. As the study included 931 participants, it had adequate sample size and statistical power to detect significant differences among the groups.

## Results

### Clinical and Demographic Data

Treatment-resistance (TRS) differed significantly (χ^2^ = 69.694; *df* = 1; *p* < 0.001) between male and female patients, since males were more frequently treatment resistant (79.6%) than females (20.4%), and the lowest number of female TRS patients (*R* = 5.4) significantly contributed to this significance. Therefore, in further analyses all TRS and non-TRS patients were subdivided according to gender (**Table 1**).

**Table 1:** The demographic and clinical data of male and female patients with schizophrenia subdivided in TRS and non-TRS groups.

	TRS (*N* = 270)		Non-TRS (*N* = 661)		
	Males (*N* = 215)	Females (*N* = 55)	Males (*N* = 330)	Females (*N* = 331)	
Age (years) (median, 25 and 75 percentile)	39 (30,45)	48 (38,58)	40 (31,49)	50 (39,57)	*H* = 106.086; *df* = 3; *p* < 0.001^∗^; Kruskal–Wallis ANOVA
Smokers, *N* (%)	155 (72.09%)	27 (49.09%)	220 (66.67%)	172 (51.96%)	χ^2^ = 198.8; *df* = 3; *p* < 0.0001^∗^; χ^2^-test
PANSS total scores (median, 25 and 75 percentile)	130 (115,143)	106 (98,124)	105 (92,123)	90 (75,102)	*H* = 324.222; *df* = 3; *p* < 0.001^∗^; Kruskal–Wallis ANOVA
PANSS positive scores (median, 25 and 75 percentile)	34 (30,39)	28 (26,32)	26 (22,33)	22 (17,26)	*H* = 300.511; *df* = 3; *p* < 0.001^∗^; Kruskal–Wallis ANOVA
PANSS negative scores (median, 25 and 75 percentile)	34 (28,37)	27 (23,30)	26 (23,31)	23 (19,26)	*H* = 248.191; *df* = 3; *p* < 0.001^∗^; Kruskal–Wallis ANOVA
PANSS general psychopathology scores (median, 25 and 75 percentile)	62 (56,69)	54 (48,63)	52 (45,59)	45 (38,51)	*H* = 279.877; *df* = 3; *p* < 0.001^∗^; Kruskal–Wallis ANOVA
Chlorpromazine equivalent doses, mg/per day (median, 25 and 75 percentile)	700 (500,1000)	500 (400,750)	500 (300,750)	400 (200,500)	*H* = 136.508; *df* = 3; *p* < 0.001^∗^; Kruskal–Wallis ANOVA

*Results are presented as medians with 25 and 75 percentile. *N* is the number of patients. ^∗^Significant differences between male and female TRS and non-TRS patients. Non-TRS, non-treatment-resistant schizophrenia; PANSS, Positive and Negative Syndrome Scale; TRS, treatment-resistant schizophrenia.*

Dunn *post hoc* analysis performed following Kruskal–Wallis ANOVA confirmed significant difference (*p* < 0.001) in age, chlorpromazine equivalent doses, PANSS total, positive, negative, and general psychopathology scores between male and female patients in TRS, as well as in non-TRS group. Moreover, there were also significant differences (*p* < 0.001) between male patients with TRS and non-TRS, as well as between female patients with TRS and non-TRS, in chlorpromazine equivalent doses, PANSS total, positive, negative, and general psychopathology scores, but not in the age.

As shown in **Table 1**, the distribution of smokers and non-smokers was also significantly different (*p* < 0.0001) between male and female patients with TRS and non-TRS. Significantly (*R* = 2.86) lower frequency of female non-smokers in non-TRS group contributed to this difference. Males smoked more often than females in both TRS (χ^2^ = 10.55; *df* = 1; *p* = 0.0012) and no-TRS group (χ^2^ = 15.27; *df* = 1; *p* < 0.0001); however, there were no significant differences between male patients with TRS and non-TRS, as well as between female patients with TRS and non-TRS.

### Genotype Analysis

In male patients with schizophrenia, in the TRS group, *COMT* rs4818 (χ^2^ = 1.333; *df* = 1; *p* = 0.248) and *COMT* rs4680 (χ^2^ = 0.929; *df* = 1; *p* = 0.335) genotypes distributions did not deviate from HWE. In male patients who were not treatment resistant (i.e., in non-TRS group), no departure from HWE was found for *COMT* rs4818 (χ^2^ = 0.447; *df* = 1; *p* = 0.503) and *COMT* rs4680 (χ^2^ = 1.492; *df* = 1; *p* = 0.222) genotypes. In female TRS patients, no significant deviation from HWE in *COMT* rs4818 (χ^2^ = 1.603; *df* = 1; *p* = 0.206) and *COMT* rs4680 (χ^2^ = 0.197; *df* = 1; *p* = 0.657) genotypes distributions was detected. Among female patients in non-TRS group, frequencies of *COMT* rs4818 (χ^2^ = 0.981; *df* = 1; *p* = 0.322) and *COMT* rs4680 (χ^2^ = 0.114; *df* = 1; *p* = 0.736) genotypes did not deviate from HWE.

There were no significant differences in the frequency of the genotypes or in the dominant or recessive model for the *COMT* rs4818 and COMT rs4680 between male patients with or without TRS (**Table 2**).

**Table 2:** The distribution of the *COMT* rs4818 and rs4680 genotypes in male patients with schizophrenia subdivided into TRS and non-TRS groups.

Male patients (*N* = 545)		TRS (*N* = 215)		Non-TRS (*N* = 330)		
		*N*	Frequency (%)	*N*	Frequency (%)	
COMT rs4818 genotypes	CC	60	27.9	83	25.2	χ^2^ = 2.315; *df* = 2;
	CG	99	46.0	171	51.8	*p* = 0.314; χ^2^-test
	GG	56	26.0	76	23.0	
C carriers	C carriers	159	74.0	254	77.0	χ^2^ = 0.911; *df* = 1;
	GG	56	26.0	76	23.0	*p* = 0.340; χ^2^-test
G carriers	G carriers	155	72.1	247	74.8	χ^2^ = 0.671; *df* = 1;
	CC	60	27.9	83	25.2	*p* = 0.413; χ^2^-test
COMT rs4680 genotypes	AA	85	39.5	119	36.1	χ^2^ = 1.741; *df* = 2;
	AG	95	44.2	167	50.6	*p* = 0.419; χ^2^-test
	GG	35	16.3	44	13.3	
A carriers	A carriers	180	83.7	286	86.7	χ^2^ = 0.645; *df* = 1;
	GG	35	16.3	44	13.3	*p* = 0.422; χ^2^-test
G carriers	G carriers	130	60.5	211	63.9	χ^2^ = 0.511; *df* = 1;
	AA	85	39.5	119	36.1	*p* = 0.475; χ^2^-test

*Results are presented as numbers and %. *N* is the number of patients. COMT, catechol-*O*-methyl-transferase; C carriers of the COMT rs4818, the combined CC and CG genotype; G carriers of the COMT rs4818, the combined GG and GC genotype; G carriers of the COMT rs4680, the combined GG and AG genotype; A carriers of the COMT rs4680, the combined AA and AG genotype; non-TRS, non-treatment-resistant schizophrenia; TRS, treatment-resistant schizophrenia.*

In female patients (**Table 3**) subdivided into TRS and non-TRS groups, significant differences were found in the frequency of the *COMT* rs4818 genotypes (CC, CG, and GG; *p* = 0.014) and in the dominant model (C carriers, i.e., CC + CG vs. GG; *p* = 0.008). The distribution of the genotypes in the recessive model (G carriers, i.e., GG + GC vs. CC; *p* = 0.043) for the *COMT* rs4818 did not differ significantly between female TRS and non-TRS groups (**Table 3**). Further analysis revealed that the C carriers were five times more likely to be in the TRS group than GG homozygotes [odds ratio (OR) (C carriers/GG) = 5.748; 95% confidence interval (CI) (1.362–24.251), *z* = 2.381; *p* = 0.017] in female patients with schizophrenia. Similar frequency of the *COMT* rs4680 genotypes (GG, GA and AA), and the genotypes in the recessive (A carriers, i.e., GA + AA vs. GG) or dominant (G carriers, i.e., GG + GA vs. AA) model was found in female TRS and non-TRS patients (**Table 3**). Although a difference in the distribution of the G carriers vs. AA homozygotes was not significant (*p* = 0.033) due to correction (*p* = 0.025), female carriers of AA genotype of the *COMT* rs4680 were slightly more frequent in TRS group than G carriers (*R* = 1.7; OR (AA homozygotes/ G carriers) = 1.917, 95% CI (1.046–3.515); *z* = 2.105; *p* = 0.035). These results showed that female AA homozygotes had almost double chance to develop TRS when compared to G carriers in female patients with schizophrenia (**Table 3**).

**Table 3:** The distribution of the *COMT* rs4818 and rs4680 genotypes in female patients with schizophrenia subdivided into TRS and non-TRS group.

Female patients (*N* = 386)		TRS (*N* = 55)		Non-TRS (*N* = 331)		
		*N*	Frequency (%)	*N*	Frequency (%)	
COMT rs4818 genotypes	CC	28	50.9	121	36.6	χ^2^ = 8.525; *df* = 2;
	CG	25	45.5	151	45.6	*p* = 0.014^∗^; χ^2^- test
	GG	2	3.6	59	17.8	
C carriers	C carriers	53	96.4	272	82.2	χ^2^ = 7.136; *df* = 1;
	GG	2	3.6	59	17.8	*p* = 0.008^∗^; χ^2^-test
G carriers	G carriers	27	49.1	210	63.4	χ^2^ = 4.100; *df* = 1;
	CC	28	50.9	121	36.6	*p* = 0.043; χ^2^-test
COMT rs4680 genotypes	AA	20	36.4	76	23.0	χ^2^ = 5.263; *df* = 2;
	AG	25	45.5	162	48.9	*p* = 0.072; χ^2^-test
	GG	10	18.2	93	28.1	
A carriers	A carriers	45	81.8	238	71.9	χ^2^ = 2.370; *df* = 1;
	GG	10	18.2	93	28.1	*p* = 0.124; χ^2^-test
G carriers	G carriers	35	63.6	255	77.0	χ^2^ = 4.534; *df* = 1;
	AA	20	36.4	76	23.0	*p* = 0.033; χ^2^-test

*Results are presented as numbers and %. *N* is the number of patients. ^∗^Significant difference in the distribution of the CC, CG, and GG genotypes; or of the C carriers and GG homozygotes, between TRS and non-TRS patients. COMT, catechol-*O*-methyl-transferase; C carriers of the COMT rs4818, the combined CC and CG genotype; G carriers of the COMT rs4818, the combined GG and GC genotype; G carriers of the COMT rs4680, the combined GG and AG genotype; A carriers of the COMT rs4680, the combined AA and AG genotype; non-TRS, non-treatment-resistant schizophrenia; TRS, treatment-resistant schizophrenia.*

### Haplotype Analysis

**FIGURE 1:**
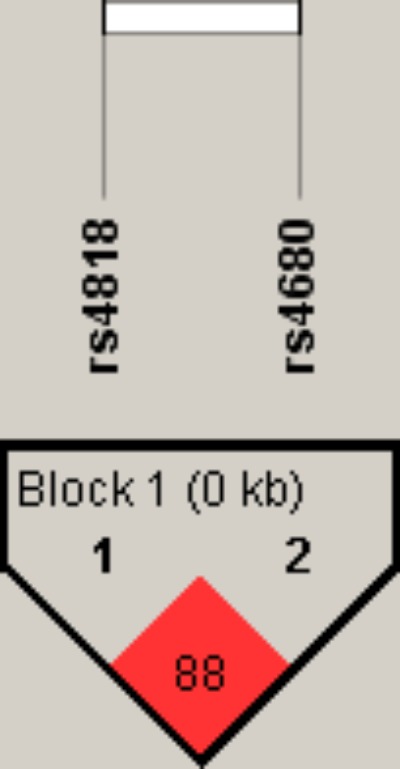
Linkage disequilibrium (LD) plot with D’ values characterizing haplotype blocks in COMT gene. The plot was generated by Haploview 4.2 software. Block definition followed the rules of solid spine of LD. D’ values multiplied by 100 are shown as a number in the diamonds.

To further examine the association of *COMT* rs4818 and rs4680 polymorphisms with TRS in male and female patients, a haplotype analysis was performed. As shown in **Figure 1**, a high degree of linkage disequilibrium (*D*′ = 0.88) was revealed for *COMT* rs4818 and rs4680 polymorphisms.

**Table 4** shows the frequencies of the *COMT* (rs4818-rs4680) haplotypes in all patients subdivided in TRS and non-TRS group. The most common haplotype was C-A, followed by G-G haplotype. There was a significant difference (*p* = 0.037) in the distribution of G-G haplotype among TRS and non-TRS patients, demonstrating that G-G haplotype was less frequently represented in TRS than in non-TRS group (**Table 4**).

**Table 4:** The distribution of *COMT* rs4680-rs4818 haplotypes in all patients with schizophrenia subdivided into TRS and non-TRS group.

Haplotype	All patients	TRS, Non-TRS	
	Frequency (%)	Frequency (%)	
C-A	48.0	49.4, 47.4	χ^2^ = 0.585; *p* = 0.444; χ^2^-test
G-G	36.3	32.7, 37.8	χ^2^ = 4.436; *p* = 0.037^∗^; χ^2^-test
C-G	13.5	14.7, 13.0	χ^2^ = 1.019: *p* = 0.313; χ^2^-test
G-A	2.2	3.2, 1.8	χ^2^ = 3.422; *p* = 0.064; χ^2^-test

*Results are presented as %. COMT, catechol-*O*-methyl-transferase; non-TRS, non-treatment-resistant schizophrenia; TRS, treatment-resistant schizophrenia. ^∗^Significant differences in the frequency of G-G haplotype between TRS and non-TRS patients.*

In the analysis of the *COMT* rs4818-rs4680 haplotypes, all patients were subdivided into carriers and non-carriers of the particular haplotype. For two of the most common haplotype (C-A and G-G) groups, subjects were additionally subdivided into “homozygotes” for the particular haplotype (carriers of the two same haplotype groups), individuals that carry only one of the examined haplotype, and non-carriers of the tested haplotype. Because of the low frequency of the “homozygotes”, for another two haplotype groups (C-G and G-A), patients were divided only into carriers and non-carriers.

Haplotype analysis showed a lack of significant difference in the frequency of the particular haplotypes between male TRS and non-TRS patients (**Table 5**).

**Table 5:** Haplotype frequencies of *COMT* rs4680 and rs4818 polymorphisms in male patients with schizophrenia subdivided according to the TRS.

Male patients (*N* = 565)		TRS (*N* = 215)		Non-TRS (*N* = 330)		
		*N*	Frequency (%)	*N*	Frequency (%)	
Haplotype C- A	C-A/C-A	52	24.2	75	22.7	χ^2^ = 2.494; *df* = 2;
	C-A/^∗^	102	47.4	178	53.9	*p* = 0.287; χ^2^-test
	^∗^/^∗^	61	28.40	77	23.3	
C-A carriers	C-A carriers	154	71.6	253	76.7	χ^2^ = 1.748; *df* = 1;
	Non-carriers	61	28.4	77	23.3	*p* = 0.186; χ^2^-test
Haplotype G-G	G-G/ G-G	30	14.0	43	13.0	χ^2^ = 1.730; *df* = 2;
	G-G/^∗^	92	42.8	160	48.5	*p* = 0.421; χ^2^-test
	^∗^/^∗^	93	43.3	127	38.5	
G-G carriers	G-G carriers	122	56.7	203	61.5	χ^2^ = 1.231; *df* = 1;
	Non-carriers	93	43.3	127	38.5	*p* = 0.267; χ^2^-test
C-G carriers	C-G carriers	49	22.8	77	23.3	χ^2^ = 0.022; *df* = 1;
	Non-carriers	166	77.2	253	76.7	*p* = 0.883; χ^2^-test
G-A carriers	G-A carriers	12	5.6	9	2.7	χ^2^ = 2.862; *df* = 1;
	Non-carriers	203	94.4	321	97.3	*p* = 0.091; χ^2^-test

*^∗^Other haplotypes. Results are presented as numbers and %. *N* is the number of patients. COMT, catechol-*O*-methyl-transferase; non-TRS, non-treatment-resistant schizophrenia; TRS, treatment-resistant schizophrenia.*

In female (**Table 6**) patients with schizophrenia, haplotype distribution differed significantly between TRS and non-TRS patients. Among female schizophrenia patients, haplotype G-G was detected less frequently in TRS group (*R* = 1.5; *p* = 0.011) compared to non-TRS group. Detailed analysis showed that female non-carriers of the haplotype G-G were 2 times more likely to be treatment resistant than female carriers of the haplotype G-G (OR (non-carriers/GG carriers) = 2.1015; 95% CI (1.179–3.743); *z* = 2.522; *p* = 0.012). Haplotype analysis confirmed genotyping results since haplotype frequency of G-G/G-G, G-G/^∗^ and ^∗^/^∗^ haplotypes between female TRS and non-TRS patients differed significantly (*p* = 0.009). Namely, presence of the G allele was “protective” against TRS. The high activity G-G/G-G haplotype was the rarest haplotype (3.6%) in female patients with TRS, followed by G-G/^∗∗^ haplotype, while carriers of any other haplotype than G-G were overrepresented (56.4%) in female patients with TRS in comparison to non-TRS female patients (**Table 6**).

**Table 6:** Haplotype frequencies of *COMT* rs4680 and rs4818 polymorphisms in female patients with schizophrenia subdivided according to the TRS.

Female patients (*N* = 386)		TRS (*N* = 55)		Non-TRS (*N* = 331)		
		*N*	Frequency (%)	*N*	Frequency (%)	
Haplotype C-A	C-A/C-A	17	30.9	71	21.5	χ^2^ = 4.198; *df* = 2;
	C-A/^∗^	28	50.9	161	48.6	*p* = 0.123; χ^2^-test
	^∗^/^∗^	10	18.2	99	29.9	
C-A carriers	C-A carriers	45	81.8	232	70.1	χ^2^ = 3.201; *df* = 1;
	Non-carriers	10	18.2	99	29.9	*p* = 0.074; χ^2^-test
Haplotype G-G	G-G/G-G	2	3.6	53	16.0	χ^2^ = 9.318; *df* = 2;
	G-G/^∗^	22	40.0	152	45.9	*p* = 0.009#; χ^2^-test
	^∗^/^∗^	31	56.4	126	38.1	
G-G carriers	G-G carriers	24	43.6	205	61.9	χ^2^ = 6.544; *df* = 1;
	Non-carriers	31	56.4	126	38.1	*p* = 0.011#; χ^2^-test
C-G carriers	C-G carriers	15	27.3	81	24.5	χ^2^ = 0.198; *df* = 1;
	Non-carriers	40	72.7	250	75.5	*p* = 0.656; χ^2^-test
G-A carriers	G-A carriers	3	5.5	8	2.4	χ^2^ = 1.572; *df* = 1;
	Non-carriers	52	94.5	323	97.6	*p* = 0.210; χ^2^-test

*^∗^Other haplotypes. Results are presented as numbers and %. *N* is the number of patients. COMT, catechol-*O*-methyl-transferase; non-TRS, non-treatment-resistant schizophrenia; TRS, treatment-resistant schizophrenia. #Significant differences in the frequency of G-G/G-G, G-G/^∗^ and ^∗^/^∗^ haplotypes between TRS and non-TRS patients.*

## Discussion

### Clinical Differences Between Patients With TRS and Non-TRS

Our findings of four times higher prevalence of TRS in male compared to female patients are in contrast to results from Danish patients (Wimberley et al., 2017) who had higher rates of TRS in female patients. However, this study used different methodology, such as the determination of TRS by a treatment-based proxy (Wimberley et al., 2017). In our study, both male and female patients with TRS had significantly higher PANSS total, positive, negative, and general psychopathology scores, as well as higher chlorpromazine equivalent doses in comparison to male and female patients in non-TRS group. This is in agreement with previous studies reporting that patients with TRS had more severe symptomatology as measured by PANSS (Moretti et al., 2018) and received higher total antipsychotic dose presented as chlorpromazine equivalents, compared to patients with non-TRS (Hotta et al., 2011; de Bartolomeis et al., 2018; Moretti et al., 2018). In the present study, the concentration of antipsychotics in plasma was not measured. While about one-third of patients with TRS had sub-therapeutic or non-detectable antipsychotic plasma levels (McCutcheon et al., 2018), non-adherence, or only partial adherence in some of our patients with TRS cannot be ruled out. However, unlike the latter article which excluded patients on long-acting antipsychotics (McCutcheon et al., 2018), in the present study about a quarter of patients received different depot antipsychotics (data not shown), which provided continuous drug delivery. Furthermore, while the majority of patients with TRS were hospitalized at the time of the assessment, antipsychotic intake was monitored by hospital stuff. Given the pronounced difference (an average of 100–200 mg/per day) in chlorpromazine equivalents between individuals with and without TRS, some of those patients might developed the antipsychotic-induced dopamine suprasensitivity psychosis (DSST). Although the presence of neurological disorders was exclusion criteria, rating scales for the assessment of movement disorders were not performed. Therefore, while subjects with pronounced extrapyramidal symptoms or tardive dyskinesia were not included, subtle movement disorders, indicative of DSST (Chouinard et al., 2017), might have gone undetected in some individuals. In the present study, the prevalence of smoking was higher in males than females, in both patients with TRS and non-TRS. This is in line with our previous data showing higher prevalence of smoking in male (64%) than in female (46%) patients with schizophrenia (Nikolac et al., 2013), and agrees with the data from general population, given that the men had higher smoking prevalence than woman in European countries, including Croatia (Gallus et al., 2014). Men with schizophrenia also had higher rates of nicotine dependence and different smoking habits compared to healthy men (Nikolac et al., 2013; Šagud et al., 2018). However, the rate of smoking in our study was similar in patients with TRS and non-TRS, which is consistent with the finding from the smaller sample of 21 patients with TRS and 20 patients with non-TRS (Mouchlianitis et al., 2016). Although patients with schizophrenia have the highest known rates of smoking (de Leon and Diaz, 2005), confirmed by the 62.8% of smokers found in schizophrenia patients in our previous study (Nedic Erjavec et al., 2017), our results suggest that treatment-resistance does not further increase this rate. However, smoking might be related to more severe clinical presentation in patients with TRS, since smokers with TRS had higher PANSS total scores and negative subscale scores, and performed significantly worse on the problem solving cognitive task, compared to TRS patients who did not smoke (Iasevoli et al., 2013). The relationship between schizophrenia and smoking is complex (Šagud et al., 2009) given that smoking status was also related to lower total PANSS scores and the PANSS general psychopathology scores in patients with schizophrenia (Nedic Erjavec et al., 2017). Smoking might contribute to treatment resistance by decreasing serum levels of clozapine and olanzapine, but the dose increase might overcome those effects (Tsuda et al., 2014).

### Gender-Related Differences in the COMT rs4680 and rs4818 Genotypic and Haplotypic Association With Treatment Resistance

Our results confirmed gender-related differences in the genotypic and haplotypic association of the *COMT* rs4680 and rs4818 and treatment resistance in patients with schizophrenia. Namely, our study revealed that: (1) in male patients with schizophrenia, there were no significant haplotypic and genotypic associations between *COMT* rs4680 and rs4818 and treatment-resistance; (2) in female patients with TRS, AA genotype carriers of the *COMT* rs4680 were nominally more frequently present compared to G carriers, whereas CC carriers of the *COMT* rs4818 were significantly more frequent than G carriers; (3) in female patients with TRS, the high activity G-G/G-G *COMT* haplotype was rare, followed by G-G/^∗∗^ haplotype, while carriers of any other than G-G haplotype were overrepresented, in comparison to female patients with non-TRS. To the best of our knowledge, this is the first report to document that the presence of high-activity (G variants) of the *COMT* rs4680 and rs4818, and *COMT* G-G/G-G haplotype, appears to be associated with lower risk of TRS in female patients with schizophrenia, while no such associations were observed in men. These findings suggest the significant, but gender-specific associations of *COMT* variants with the development of treatment-resistance in schizophrenia.

### Gender-Related Association Between the COMT rs4680 Genotype and Treatment Resistance

Gender differences were previously noticed in COMT activity (Chen et al., 2004) as well as in distribution of the *COMT* rs4680 genotypes in healthy individuals (Gurvich and Rossell, 2015; El-Hage et al., 2017) and patients with schizophrenia (Bollettini et al., 2017). There is also evidence of distinct, sex-dependent brain changes related to *COMT* rs4680 polymorphism (Bollettini et al., 2017; El-Hage et al., 2017). Female patients with schizophrenia, carriers of the *COMT* rs4680 AA genotype, had smaller volumes of caudate, putamen, and pallidum, while male patients, homozygous for the Met allele showed higher or similar subcortical volumes compared to other groups (Bollettini et al., 2017). In healthy volunteers, male GG homozygotes had higher white matter integrity compared to A carriers, whereas no differences were observed in females (El-Hage et al., 2017). In healthy women, carriers of the *COMT* rs4680 AA genotype had reduced and GG genotype carriers had superior cognitive flexibility, whereas in men no association with cognition was found (Gurvich and Rossell, 2015). Those studies suggested that AA homozygosity, specifically in women, might be adversely associated with cognitive functioning (Gurvich and Rossell, 2015) and subcortical brain volumes (Bollettini et al., 2017), which could be also related to the association of *COMT* rs4680 polymorphism with treatment-resistance observed only in female patients with schizophrenia in the present study. On the other hand, some studies have not observed associations of *COMT* rs4680 polymorphism with age or gender (Walder et al., 2010; Collip et al., 2011; Armbruster et al., 2012).

Four studies have addressed the association between *COMT* rs4680 and TRS so far. Two of them have included only patients with TRS (Bosia et al., 2015; Rajagopal et al., 2018) whereas two other trials included patients with both TRS and non-TRS (Inada et al., 2003; Terzić et al., 2016). The discrepancies across the studies might arise from sex-differences, ethnic differences, diversities in the populations studied, limited power and small sample sizes, as well as different methodology such as the various definitions of treatment-resistance and measurements of psychopathology. In contrast to our results, TRS patients, carriers of the AA or GA genotypes of the *COMT* rs4680, but who were also carriers of one or two DRD4 120-bp alleles (120/240 and 120/120), experienced better response to clozapine than TRS patients, carriers of the GG genotype (Rajagopal et al., 2018). This study included 93 TRS patients of South Indian ethnicity, but did not divide patients according to gender. Corresponding to our results in male patients with schizophrenia, *COMT* rs4680 genotypes were not associated with better response to clozapine in TRS (Rajagopal et al., 2018). Since in our study the presence of the A allele was associated with TRS in female but not in male patients, either different number of patients, the fact that we did not evaluate gene-gene interaction with the DRD4 120-bp alleles, or ethnic differences (Rajagopal et al., 2018), might explain these different results. In line with the results from the study evaluating 107 treatment-resistant Italian patients (Bosia et al., 2015), carriers of the *COMT* rs4680 GG genotype have shown better response to clozapine (in reducing negative symptoms), compared to patients with the GA and AA genotypes (Bosia et al., 2015). In our study, this slight association was observed only in female but not in male patients. Since the cited study (Bosia et al., 2015) did not evaluate gender specific association with TRS, and included much smaller sample, we might speculate that this non-significant association (due to correction) of the AA genotype with TRS might be presumably related to female gender. Our results on the link between the AA genotype and TRS in female patients with schizophrenia also agree with the data from the 100 Japanese patients with schizophrenia (Inada et al., 2003). In this study, patients with TRS had marginally higher frequency of the A variant of *COMT* rs4680 polymorphism, and the odds ratio for the AA genotype in TRS was 4.392 (Inada et al., 2003). Patients with the *COMT* rs4680 AA genotype also received higher chlorpromazine equivalent doses compared to carriers of the GA and GG genotypes (Inada et al., 2003). Although the sample size was small (only eight patients had *COMT* rs4680 AA genotype), and patients were not evaluated according to gender (Inada et al., 2003), these results are in line with our female data. In agreement with our data in males, but in contrast to data obtained in female patients with or without TRS, no difference in the *COMT* rs4680 genotype frequency was detected in 138 patients: 44 treatment-resistant and 94 treatment-responsive patients from Slovenia, who were not divided by gender (Terzić et al., 2016).

#### Gender-Related Association Between the COMT rs4818 Genotype and Treatment Resistance

While *COMT* rs4680 is among the most frequently investigated polymorphisms in treatment response to psychotropic drugs, only a few studies investigated *COMT* rs4818 polymorphism and the response to antipsychotics (Gupta et al., 2009; Xu et al., 2015; Shi et al., 2017). In our study the presence of the CC genotype of the *COMT* rs4818 in female, but not in male group of patients with TRS, was found significantly more frequently than the presence of the GC and GG genotypes. In line with our findings, in the large Shanghai cohort of 995 Chinese patients with schizophrenia, C carriers of the *COMT* rs4818 had more frequently poor response to quetiapine (Xu et al., 2015). The association between *COMT* rs4818 and treatment response to risperidone was also reported in 288 Shanghai patients with schizophrenia (Shi et al., 2017), but opposed to our and their previous (Xu et al., 2015) results, the G/C allele frequency was similar between good and poor responders (Shi et al., 2017).

### Gender-Related Association Between the COMT rs4680 and rs4818 Haplotype and TRS

The findings of the present study that carriers of the high activity G-G/G-G haplotype were more frequently observed in non-TRS female group, whereas female carriers of any other haplotype than G-G were overrepresented in the group of patients with TRS, suggest that presence of the G allele might be associated with decreased risk of TRS. This association was also confirmed in Shanghai cohort (Xu et al., 2015). In contrast to our data, among 117 patients with schizophrenia of the southern Indian origin, the *COMT* haplotype C-A (rs4818-r4680) was observed more often in responders to risperidone, compared to non-responders (Gupta et al., 2009). However, these data were not analyzed by gender, and the treatment-response was defined only by the clinical global impression scale (Gupta et al., 2009).

Our results showed that presence of the high activity (i.e., G variants) of the *COMT* was associated with lower risk of TRS in female patients. Likewise, negative symptoms of schizophrenia were less severe in female patients carrying the high activity *COMT* variants (rs740603 (G)-rs4818 (G) haplotype), but this effect was not observed in male patients with schizophrenia (Li et al., 2012). If this finding will be confirmed in larger studies and meta-analyses, women with schizophrenia who are carriers of the *COMT* rs4680 and rs4818 low-activity haplotypes, might require different treatment approach, such as early clozapine initiation (Siskind et al., 2017), clozapine augmentation with different antiepileptic drugs (Zheng et al., 2017), or electroconvulsive therapy (ECT) (Vuksan et al., 2018).

### The Association Between the High-Activity COMT Haplotype and Treatment Response in Female Patients With Schizophrenia: Possible Explanations

COMT activity plays a key role in the regulation of dopamine activity in PFC, while its role in the regulation of striatal dopamine turnover is less important due to higher abundance of dopamine transporter in this region (Bilder et al., 2004). In line with this hypothesis, in healthy subjects, *COMT* rs4680 GG carriers had lower dopamine tone in cortical and limbic regions, compared to carriers of the GA or AA genotypes, while no changes of dopamine tone were detected in the striatal regions (Slifstein et al., 2008). However, striatal dopamine turnover seems to be altered in patients with TRS (Kim et al., 2017). While patients with schizophrenia generally exhibited elevated striatal dopamine synthesis capacity compared to healthy controls (Fusar-Poli and Meyer-Lindenberg, 2013), individuals suffering from TRS had lower capacity of dopamine synthesis in striatum (Kim et al., 2017). This finding was observed in TRS patients treated with clozapine (Kim et al., 2017) and in patients treated with other antipsychotics (Demjaha et al., 2012), in comparison to patients who responded to antipsychotic treatment. Although COMT has a minor role in metabolizing striatal dopamine, modifications of COMT activity may affect dopamine signaling also in the striatum, as shown by the data from animal models (Simpson et al., 2014; Tammimaki et al., 2016), and from some (Boot et al., 2011) but not all (Slifstein et al., 2008) human reports. Considering that compensatory mechanisms might influence dopamine function in striatum (Simpson et al., 2014), the relationship between prefrontal dopamine availability, modulated by *COMT* rs4680, and striatal dopamine tone (Bilder et al., 2004; Ceaser et al., 2013) was proposed. In post-mortem brain samples of individuals without psychiatric disorders, carriers of the *COMT* rs4680 GG genotype had greater expression of tyrosine hydroxylase mRNA in mesencephalic dopamine neurons than GA genotype carriers, particularly in neuronal populations that project to the striatum, suggesting higher dopamine synthesis in striatal regions of GG homozygotes (Akil et al., 2003).

Although these studies did not account the possible gender differences (Akil et al., 2003; Slifstein et al., 2008), we might speculate that females with AA genotype of the *COMT* rs4680 might have had higher prefrontal and compensatory lower striatal dopamine levels, which were reported in patients with TRS (Kim et al., 2017). The dopamine synthesis capacity in striatum was proposed as a biomarker for TRS (Kim et al., 2017). In the presence of higher dopamine stimulation from PFC, such as in the *COMT* rs4680 AA homozygous subjects, dopamine release might decrease in striatum in an attempt to protect the brain from excessive dopaminergic stimulation, and this mechanism might be related to treatment-resistance. According to our findings, this hypothesis might be only relevant for female patients with schizophrenia. Given the COMT-inhibiting properties of estradiol (McDermott et al., 2015), decreased COMT activity in women (Chen et al., 2004), and large sex-differences in dopaminergic cortical pathways in preclinical model (Kritzer and Creutz, 2008), it could be hypothesized that women have higher PFC dopamine levels than men, carrying the same *COMT* genotype. Such greater dopaminergic stimulation in females could lead to a gender-specific hyperdopaminergic overdrive in PFC, and consequently to decreased dopamine levels in striatum, which might eventually predispose women to TRS. This proposal fits the presumption that some patients do not respond to treatment because they do not exhibit elevated dopamine input in striatum (Demjaha et al., 2012; Kim et al., 2017), while increased dopamine stimulation in striatum is the target for antipsychotic drugs (Fusar-Poli and Meyer-Lindenberg, 2013). However, the current study did not measure dopamine levels.

The evidence of sexual dimorphism of *COMT* gene is still inconclusive, but continues to accumulate (Harrison and Tunbridge, 2008). The findings regarding sex-dependent associations of COMT with various clinical and biological features have been reported in diverse populations (Rybakowski et al., 2006; Lang et al., 2007; Chen C. et al., 2011; Jacobs and D’Esposito, 2011; Klebe et al., 2013; Koike et al., 2018), as well as in preclinical trials (Gogos et al., 1998; Laatikainen et al., 2013; Sannino et al., 2015). In general, sexually dimorphic effects of *COMT* gene variations are complex, and range from robust to subtle, depending on the parameters, which were measured.

Great amount of the data demonstrating gender differences in association of *COMT* gene with various personality traits, phenotypes, cognitive domains and behaviors, came from studies that enrolled healthy individuals (Eley et al., 2003; Enoch et al., 2003; Olsson et al., 2005; Stein et al., 2005; Kim et al., 2006; Barnett et al., 2007; Lang et al., 2007; de Castro-Catala et al., 2015; Costa et al., 2016). However, sexual dimorphism of *COMT* gene has been also reported in various neuropsychiatric disorders such as anxiety disorders, depression, attention deficit hyperactivity disorder, and obsessive-compulsive disorder (Karayiorgou et al., 1997, 1999; Domschke et al., 2004, 2007; Poyurovsky et al., 2005; Denys et al., 2006; Rothe et al., 2006; Pooley et al., 2007; Cao et al., 2014; Akutagava-Martins et al., 2016). In addition, our results are in line with other studies demonstrating sex-specific associations of *COMT* variants in patients with schizophrenia (Shifman et al., 2002; Dempster et al., 2006; Rybakowski et al., 2006; Lee and Kim, 2011). All these data suggest that *COMT* gene variations might contribute to the sex differences in brain function and structure (Domschke et al., 2012; White et al., 2014; Sannino et al., 2015, 2017; Elton et al., 2017), and consequently result in sexual dimorphism in the predisposition to various neuropsychiatric disorders. Sex-specific effects of COMT gene are usually attributed to transcriptional regulation by estrogens, and it has been suggested that reciprocal and partly genotype-influenced interactions between COMT and estrogens may be relevant to sexual dimorphism (Harrison and Tunbridge, 2008). Although the biological reason why COMT association is sex-specific is still not clear, it has been hypothesized that COMT genotype may modulate the role of estrogens in brain function and dysfunction (Seeman, 1997), while estrogens affect COMT activity and its pathophysiological consequences by influencing the COMT gene expression (Harrison and Tunbridge, 2008). However, additional mechanisms are also possible. Nevertheless, it is known from various studies that sex differences in the genetic architecture of many human traits and psychiatric disorders are common. Namely, in addition to COMT, sexually dimorphic genetic associations with psychiatric phenotypes have been reported also for other autosomal genes such as HTR2A (Enoch et al., 2001), MTHFR (Sazci et al., 2005; Kempisty et al., 2006), and AC7 (Hines et al., 2006). Therefore, our findings reporting sex-specific associations of COMT variants with the development of treatment-resistance in schizophrenia also contribute to the knowledge in this field.

### Limitations of the Study

Limitation of the study is the fact that treatment-resistance in schizophrenia patients was determined retrospectively. Another limitation of this investigation is the lower number of female then male patients, which resulted in a limited representation of some of the genotypes after the gender-stratified analysis was conducted. Therefore, the obtained results require replication in a larger sample.

## Conclusion

Our findings reveal complex and gender-dependent genotypic and haplotypic associations between *COMT* rs4680 and rs4818 and TRS. In males with schizophrenia, treatment-resistance was not associated with the *COMT* rs4818 and rs4680 genotypes or haplotypes. In contrast, as far as we are aware, this is the first study to show that in female patients with schizophrenia, the presence of high-activity (G variants) of the *COMT* rs4680 and rs4818 polymorphisms, and the presence of the G-G/G-G haplotype, is associated with the lower risk of TRS. These findings extend previously reported gender-related association of *COMT* rs4680 variants and TRS, and for the first time detect gender-dependent association of *COMT* rs4818 polymorphism with treatment-resistance in patients with schizophrenia. Accordingly, we might speculate that determination of the *COMT* rs4680 and rs4818 genotypes and haplotypes early in the course of treatment might help in the prediction of the treatment-resistance in female patients with schizophrenia. However, those findings must be interpreted with caution, given that the number of included females was substantially lower than the number of males. Therefore, investigation of the genotypic and haplotypic association between *COMT* SNPs rs4680 and rs4818 and TRS in the larger sample of females with schizophrenia is warranted.

## Author Contributions

MS did the research idea, patient recruitment, data collection, assessment by rating scales, organization of blood sampling, data interpretation, article preparation, and final draft approval. LT did experimental work, processing of blood samples, DNA isolation, genotyping, statistical analysis, and final draft approval. SU did patient recruitment, data collection, assessment by rating scales, organization of blood sampling, and final draft approval. MNP, MK, and GNE did experimental work, processing of blood samples, DNA isolation, genotyping, and final draft approval. MZ did patients recruitment, data collection, assessment by rating scales, organization of blood sampling, and final draft approval. OK, BVC, AMP, and NM did patient recruitment, data collection, assessment by rating scales, and final draft approval. DSS did experimental work, collection of blood samples, DNA isolation, genotyping, proof reading, and final draft approval. IR did data collection, and final draft approval. NP did design of the study, data analysis and interpretation, article preparation, and final draft approval.

## Conflict of Interest Statement

The authors declare that the research was conducted in the absence of any commercial or financial relationships that could be construed as a potential conflict of interest.
